# Evaluation of phenyl-propanedione on yellowing and chemical-mechanical properties of experimental dental resin-based materials

**DOI:** 10.1590/1678-775720160058

**Published:** 2016

**Authors:** Dayane Carvalho Ramos Salles de OLIVEIRA, Eduardo José SOUZA-JUNIOR, Adam DOBSON, Ana Rosa Costa CORRER, William Cunha BRANDT, Mário Alexandre Coelho SINHORETI

**Affiliations:** 1 - Universidade Estadual de Campinas, Faculdade de Odontologia de Piracicaba, Departamento de Odontologia Restauradora, Piracicaba, SP, Brasil.; 2 - Oregon Health & Science University, Department of Biomaterials, Portland, Oregon, United States of America.; 3 - Universidade de Santo Amaro, Departamento de Implantodontia, São Paulo, SP, Brasil.

**Keywords:** Dental adhesive, Dental curing lights, Dental photoinitiators, Physical and chemical properties

## Abstract

**Objective:**

To evaluate the influence of phenyl-propanedione on yellowing and chemical-mechanical properties of experimental resin-based materials photoactivated using different light curing units (LCUs).

**Material and Methods:**

Experimental resin-based materials with the same organic matrix (60:40 wt% BisGMA:TEGDMA) were mechanically blended using a centrifugal mixing device. To this blend, different photoinitiator systems were added in equimolar concentrations with aliphatic amine doubled by wt%: 0.4 wt% CQ; 0.38 wt% PPD; or 0.2 wt% CQ and 0.19 wt% PPD. The degree of conversion (DC), flexural strength (FS), Young’s modulus (YM), Knoop hardness (KNH), crosslinking density (CLD), and yellowing (Y) were evaluated (n=10). All samples were light cured with the following LCUs: a halogen lamp (XL 2500), a monowave LED (Radii), or a polywave LED (Valo) with 16 J/cm_2_. The results were analysed by two-way ANOVA and Tukey’s test (α=0.05).

**Results:**

No statistical differences were found between the different photoinitiator systems to KNH, CLS, FS, and YM properties (p≥0.05). PPD/CQ association showed the higher DC values compared with CQ and PPD isolated systems when photoactivated by a polywave LED (p≤0.05). Y values were highest for the CQ compared with the PPD systems (p≤0.05).

**Conclusion:**

PPD isolated system promoted similar chemical and mechanical properties and less yellowing compared with the CQ isolated system, regardless of the LCU used.

## INTRODUCTION

Camphorquinone (CQ) is the most widely and successfully used photoinitiator in dental resin materials^6^. Despite their high clinical acceptance, photoinitiator systems based on CQ are responsible for a yellowish colour in resin-based materials^2^ ^,^ ^6^ ^,^ ^13^.

Alternative photoinitiator systems have been suggested to substitute CQ in dental materials in order to reduce this yellowing effect, especially in resin-based materials to bleached teeth^2^ ^,^ ^6^ ^,^ ^13^ ^,^ ^16^. On the other hand, alternative photoinitiators systems for resin materials must not only have acceptable initial and long-term esthetical appearance, but also appropriated mechanical properties.

Phenyl-propanedione (PPD) is suggested as an effective alternative photoinitiator in order to reduce this yellowing^5^ ^,^ ^6^. As a Norrish type I photoinitiator, PPD reacts by photolysis, in which the cleavage of the C-C bond between the carbonyls functional groups of its molecule leads to the formation of free radicals starting the polymerization. However, PPD can also react via a co-initiator, since it bears the same diketone group as camphorquinone. Then, radicals derived from the amine-based co-initiator H-transfer are responsible for starting the polymerization^12^.

Many studies have evaluated the chemical and mechanical properties of resin-based materials associated with alternative photoinitiators, such as PPD, diphenyl(2,4,6-trimethylbenzoyl)phosphine oxide (TPO), and phenylbis(2,4,6-trimethylbenzoyl)phosphine oxide (BAPO), showing similar or superior performance compared with the CQ systems^1^ ^,^ ^4^ ^,^ ^17^. PPD is an alternative photoinitiator that shows reduced yellowing compared with the CQ systems, but its chemical and mechanical properties still need to be further evaluated^1^ ^,^ ^17^.

Unlike CQ, the absorption peak of PPD is in the near UV region (UVA) and extends slightly into the visible wavelength. Thus, it could be considered a better UV initiator than an efficient visible light photoinitiator^6^. However, some studies have shown that PPD produces similar degree of conversion compared with the CQ systems when a halogen light is used for photoactivation^3^ ^,^ ^8^ ^,^ ^14^ ^,^ ^15^. But its efficiency with different light-curing units (LCUs) for photoactivation of these resins still needs to be evaluated^5^.

The aim of the this study was to evaluate the yellowing (Y) and the chemical-mechanical properties, such as Knoop hardness (KNH), crosslinking density (CLD), degree of conversion (DC), flexural resistance (FR), and Young’s modulus (EM) of resin materials containing PPD in its composition compared with those containing CQ, photoactivated by different LCUs. The alternative hypotheses tested were as follows:

(i) PPD-based resins will promote similar or superior chemical-mechanical properties, but less yellowing compared with the CQ-based resins;

(ii) Broadband spectrum units, such as the halogen light or the polywave LED, will promote superior chemical properties for the PPD-based resins compared with the narrowed monowave LED; (iii) Broadband spectrum units, such as the halogen light or the polywave LED, will promote superior mechanical properties for the PPD-based resins compared with the narrowed monowave LED.

## MATERIAL AND METHODS

### Resin blends

Experimental resins were mechanically blended using a centrifugal mixing device SpeedMixer DAC 150.1 FVZ- K (Hauschild Engineering; Hamm, North Rhine-Westphalia, Germany) with the same organic matrix based on 60 wt% BisGMA and 40 wt% TEGDMA. To this resin, blend equimolar photoinitiator concentration were added with twice the concentration by wt% aliphatic amine, DMAEMA (Sigma-Aldrich Inc; St. Louis, MO, USA), and the following photoinitiator wt%: 0.4 wt% camphorquinone (Sigma-Aldrich Inc; St Louis, MO, USA); 0.36 wt% phenyl-propanedione (Sigma-Aldrich Inc; St Louis, MO, USA), or both in molar concentration, 0.2 wt% CQ and 0.18 wt% PPD.

### Light-curing unit evaluation

The mean and maximum radiant emittance (mW/cm^2^) and radiant exposure (J/cm^2^) according to the different wavelength ranges of each light curing unit, XL 2500 (3M/ESPE; St. Paul, MN, USA), Radii (SDI, Bayswater; Victoria, Australia), and Valo Cordless (Ultradent; South Jordan, UT, USA) were evaluated using a MARC Resin Calibrator spectrophotometer (BlueLight Analytics; Nova Scotia, Canada).

### Absorption spectrophotometric analysis

A solution of each photoinitiator was prepared using 1mL of >99.5% ethanol (Sigma-Aldrich, St. Louis, MO, USA). The solution absorption spectrophotometric analysis was determined in the 200–600 nm range using a U-2425 UV–Vis spectrophotometer (Hitachi High-Technologies; Chiyoda, Tokyo, Japan). The spectra were collected using a quartz cell with a path length of 1 cm.

### Degree of Conversion (DC)

The DC for each resin was measured using Fourier transform infrared spectroscopy (FTIR) coupled to an attenuated total reflectance (ATR), Spectrum 100 (PerkinElmer; Waltham, MA, USA). Rectangular bar-shaped specimens (10 mm x 2 mm, 1 mm thick) were photoactivated through Mylar strip using one of the light-curing units tested with 16 J/cm^2^ energy dose (n=10). The transmission spectra were recorded using 16 scans at a resolution of 1 cm^-1^ for each uncured and post-cured sample respectively. The number of remaining uncovered carbon double bonds were calculated by comparing the percentage of aliphatic C=C (vinyl) absorption (1638 cm^-1^) with aromatic C=C absorption (1608 cm^-1^) between post-cured and uncured samples, in which the aromatic double bond stretching bands remain constant during polymerization reaction and serve as an internal standard. The DC for each resin was calculated by subtracting the percentage of aliphatic double bonds present from 100%, according to the following equation:

DC (%) = {1-(Xa/Ya)/(Xb/Yb)}×100, where Xa (post-cured) and Xb (uncured) represent the areas under the bands of the polymerizable aliphatic double bonds, and Ya (post-cured) and Yb (uncured) represent the areas under the bands of aromatic double bonds.

### Flexural strength (FS) and Young’s modulus (YM)

The same specimens tested for DC were used to measure the FS and YM using a universal testing machine, model 4411 (Instron; Canton, MA, USA) in a three-point bending design (span between supports of 6.0 mm; crosshead speed of 0.5 mm/min until failure).

### Knoop hardness (KNH)

Cylindrical specimens (2 mm diameter, 2 mm thick, n=10) were used to measure KNH taken on top and bottom surfaces using a Knoop hardness meter, HMV-2 (Shimadzu; Chiyoda-ku, Tokyo, Japan), under a 0.49 N load (equivalent to 50 Kgf) for 15 s. Five readings were performed for each surface, and the mean was recorded as the KNH values (Kgf/mm^2^). The same specimens were immediately used to measure Y.

### Yellowing evaluation

Yellowing measurements of each specimen performed for KNH analysis were taken with a D65 illuminant over white (CIE L*=91.1, a*=1.2 and b*=-3.4, Y=78.8) background using a pre-calibrated spectrophotometer, CM-700d (Konica Minolta; Chiyoda-ku, Tokyo, Japan) with a diameter tip of 4 mm. The tip of the spectrophotometer was placed in the middle of each specimen using a Teflon jig and three measurements were taken of each specimen. The yellowing was determined by b* axis coordinate parameter value, in which, +b* = yellow and −b* = blue.

### Cross-linking density (CLD)

After colour measurements, the initial readings of KNH were recorded as the initial KNH number (KNH_1_) for each specimen. Then, the specimens were stored in 100% ethanol for 24 h at room temperature, and a second hardness measurement was recorded as KNH_2_. The CLD was estimated by the softening effect promoted by the ethanol with hardness decrease calculating the percentage decrease of KNH^5^.

### Statistical analysis

Data were submitted to the Shapiro-Wilk test for normality distribution verification and to the Levene’s test for homogeneity of variances. A two-way analysis of variance was used for statistical analyses of DC, FS, YM, KNH, CLD, and Δb* values. Tukey’s test was applied for multiple comparisons (p=0.05) between the different photoinitiators and light-curing units tested. All statistical analyses were performed using Stata/MP 13.0 (StataCorp; College station, TX, USA).

## RESULTS

**Figure 1:**
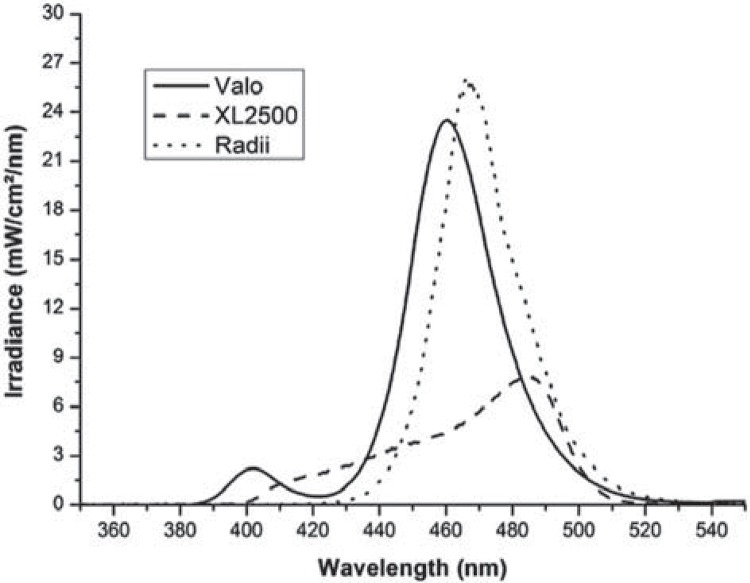
Absolute irradiance (mW/cm2/nm) x wavelength (nm) for each light-curing unit tested

**Figure 2:**
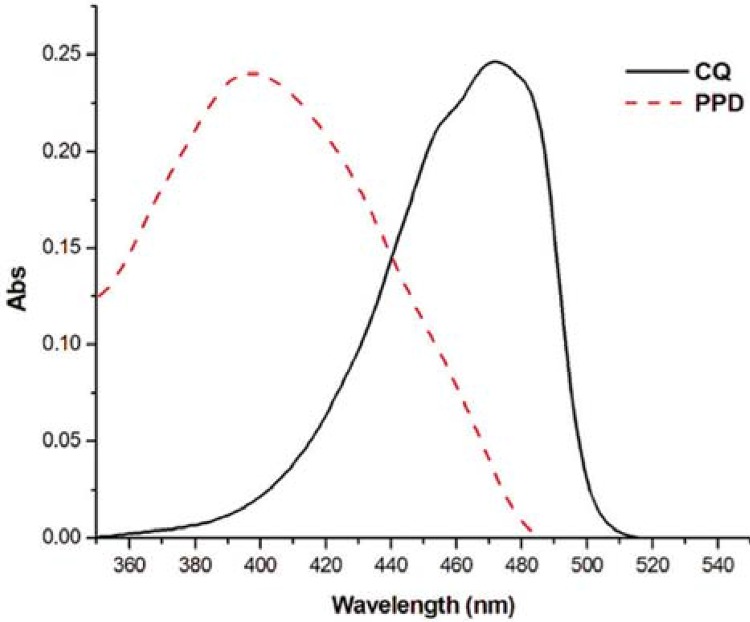
Absolute absorbance x wavelength (nm) for each photoinitiator

The LCUs wavelengths and the spectra of the photoinitiators used in this study are shown in Figures 1 and 2 respectively. Radii showed the highest spectral irradiance values (25.5 mW/cm^2^/nm) at the 465 nm emission peak, whereas Valo presented 23 mW/cm^2^/nm at the 460 nm emission peak. The halogen lamp XL 2500 presented the lowest spectral irradiance values (7.6 mW/cm^2^/nm) at the 485 nm emission peak. The light absorption analysis of dental photoinitiators showed that CQ exhibited absorption centred in the blue region of the light spectrum, with Absmax at 470 nm and ελmax 50 L/mol.cm while PPD initiates the curve in the UV region, with Absmax at 398 nm and ελmax 101 L/mol.cm, and extends the absorption curve into the visible region.

Tables 1 [Table t2] [Table t3]-[Table t4] 6 show the mean values and standard deviations for DC (%), FS (MPa), YM (GPa), KNH (Kgf/mm2), Y, and CLD (%) respectively. As it can be observed, no statistical differences were found between the different photoinitiator systems to KNH, CLS, FS, and YM properties (p≥0.05). However, PPD/CQ association showed the higher DC values compared with CQ and PPD isolated systems when photoactivated by a polywave LED (p≤0.05) and Y values were highest for the CQ compared with the PPD systems (p≤0.05).

**Table 1:** Mean DC value (%) and standard error (±) provided for each of the photoinitiator systems

Photoinitiator	Light Curing Unit		
	Radii-Cal	Valo	XL 2500
CQ	76.4 (4.1)^Aa^	79.3 (4.3)^Ab^	78.1 (2.1)^Aa^
CQ/PPD	75.4 (2.6)^Ba^	87.5 (3.2)^Aa^	79.3 (5.0)^Ba^
PPD	75.7 (4.1)^Ba^	82.8 (3.6)^Aab^	77.7 (3.9)^Ba^

Means followed by different capital letters in the same line and small letters in the same column were significantly different (p<0.05)

**Table 2 t2:** Mean FS value (MPa) and standard error (±) provided for each of the photoinitiator systems

Photoinitiator	Light Curing Unit		
	Radii-Cal	Valo	XL 2500
CQ	165.2 (30.6)^Aa^	158.4 (13.4)^Aa^	126.7 (13.4)^Ba^
CQ/PPD	174.6 (24.6)^Aa^	174.8 (20.0)^Aa^	134.1 (15.2)^Ba^
PPD	168.7 (25.1)^Aa^	166.7 (32.1)^Aa^	103.8 (20.9)^Ba^

Means followed by different capital letters in the same line and small letters in the same column were significantly different (p<0.05)

**Table 3 t3:** Mean YM value (GPa) and standard error (±) provided for each of the photoinitiator systems

Photoinitiator	Light Curing Unit		
	Radii-Cal	Valo	XL 2500
CQ	3.60 (0.77)^Aa^	3.74 (0.50)^Aa^	1.93 (0.22)^Ba^
CQ/PPD	3.74 (0.93)^Aa^	3.43 (0.53)^Aa^	1.83 (0.35)^Ba^
PPD	4.14 (0.43)^Aa^	3.73 (0.70)^Aa^	1.45 (0.39)^Ba^

Means followed by different capital letters in the same line and small letters in the same column were significantly different (p<0.05)

**Table 4 t4:** Mean KNH value (Kgf/mm2) and standard error (±) provided for each of the photoinitiator systems

Photoinitiator	Light Curing Unit		
	Radii	Valo	XL 2500
CQ	19.9 (4.1)^Aa^	23.5 (7.7)^Aa^	23.6 (6.9)^Aa^
CQ/PPD	20.9 (3.3)^ABa^	28.1 (6.7)^Aa^	16.4 (3.7)^Ba^
PPD	14.8 (4.7)^Aa^	23.0 (4.2)^Aa^	16.2 (4.5)^Aa^

Means followed by different capital letters in the same line and small letters in the same column were significantly different (p<0.05)

**Table 6:** Mean CLD value (%) and standard error (±) provided for each of the photoinitiator systems

Photoinitiator	Light Curing Unit		
	Radii	Valo	XL 2500
CQ	86.0 (7.0)^Aa^	88.2 (3.3)^Aa^	90.7 (3.7)^Aa^
CQ/PPD	85.0 (5.8)^Aa^	87.9 (3.4)^Aa^	87.3 (3.6)^Aa^
PPD	87.0 (6.4)^Aa^	89.6 (3.1)^Aa^	89.2 (3.1)^Aa^

Means followed by different capital letters in the same line and small letters in the same column were significantly different (p<0.05)

## DISCUSSION

The first alternative hypothesis that PPD-based resins will promote similar or superior chemical-mechanical properties but less yellowing compared with the CQ-based resins, could not be rejected. There were no significant differences in KNH, CLD, FS, and YM between the photoinitiator systems (p≥0.05), and PPD also promoted similar or higher DC and lower yellowing after curing compared with the CQ system, as shown in Tables 1 and 5 respectively.

**Table 5:** Mean Y value and standard deviation (±) provided for each of the photoinitiator systems

Photoinitiator	Light Curing Unit		
	Radii	Valo	XL 2500
CQ	+4.17 (0.20)^Ba^	+5.3 (0.58)^Aa^	+4.34 (0.14)^Ba^
CQ/PPD	+3.94 (0.09)^Cb^	+5.09 (0.19)^Aa^	+4.23 (0.11)^Ba^
PPD	+3.48 (0.17)^Bc^	+4.78 (0.11)^Ab^	+4.08 (0.12)^Bb^

Means followed by different capital letters in the same line and small letters in the same column were significantly different (p<0.05)

PPD has been studied as an alternative photoinitiator in order to decrease yellowing caused by CQ because of its less yellowish colour in comparison with CQ^2^ ^,^ ^5^ ^,^ ^6^ ^,^ ^14^ ^,^ ^15^. Also, as a Norrish type I photoinitiator, PPD does not require a yellowed coloured co-initiator, such as tertiary amines, to generate free radicals to start the polymerization^12^. Many studies showed that PPD reduced the Y compared with CQ and promoted similar hardness to CQ^2^ ^,^ ^6^ ^,^ ^14^. As observed in this study, PPD was able to reduce Y and also promote similar or superior chemical and mechanical properties compared with CQ.

The second alternative hypothesis that the broadband units, such as the halogen light or the polywave LED, will promote superior chemical properties for the PPD-based resins compared with the narrowed monowave LED could not be rejected, since superior DC was found in PPD-based resins compared with CQ-based systems only when photoactivated by the polywave LED. The highest DC for PPD-based systems was achieved using the polywave LED, as observed in Table 1. Unlike CQ, the absorption peak of PPD is near the UV region (UVA) (Figure 1), thus the violet spectrum irradiation by polywave LED promoted more efficient photoactivation of this alternative photoinitiator^3^ ^,^ ^6^ ^,^ ^8^ ^,^ ^15^ ^-^ ^17^, explaining the higher DC when proper spectrum emission was used for photoactivation^9^ ^,^ ^10^ ^,^ ^11^ ^,^ ^13^. The third alternative hypothesis that the broadband units, such as the halogen light or the polywave LED, will promote superior mechanical properties for the PPD-based resins compared with the narrowed monowave LED, however, was rejected. Similarities in chemical and mechanical properties between CQ and PPD formulations are explained by similarities in DC and CLD achieved in these photoinitiator systems^3^ ^,^ ^6^. As observed in this study, even when higher DC was achieved by PPD isolated system photoactivated with the polywave LED, no differences in the CLD were found. The CLD increases as the polymerization reactions increase the polymer chains. Therefore, the CLD play essential roles in mechanical properties development in comparison with the DC^7^. However, despite no improvements in mechanical properties were found for PPD-based resins in comparison with CQ, CLD similarity regarding the higher DC might be promising for filled resin materials. Since volumetric shrinkage occurs simultaneously with the increase in the CLD of the polymer^7^, PPD might reduce Y in filled resin materials without compromising marginal adaption of direct restorations. Thus, according to the findings of this study, it was possible to conclude that PPD is a promising alternative photoinitiator compared with CQ, since it reduced yellowing without compromising chemical or mechanical properties of the resins, regardless of the LCU used. PPD increased the degree of conversion when the polywave LED was used as the LCU, but no significant difference was found for CLD. Then, further studies are necessary to evaluate PPD addition to resin blends with filler addition, and polymerization shrinkage should be evaluated to determine if the increase in DC could promote less Y without affecting the marginal adaptation of direct restorations.

## CONCLUSION

According to the findings of this study, it was possible to conclude that PPD promoted similar chemical and mechanical properties and less yellowing on resins compared with the CQ-system, regardless of the LCU used.
